# Metal-Enhanced Fluorescence of Chlorophylls in Light-Harvesting Complexes Coupled to Silver Nanowires

**DOI:** 10.1155/2013/670412

**Published:** 2013-03-04

**Authors:** Dorota Kowalska, Bartosz Krajnik, Maria Olejnik, Magdalena Twardowska, Nikodem Czechowski, Eckhard Hofmann, Sebastian Mackowski

**Affiliations:** ^1^Optics of Hybrid Nanostructures Group, Institute of Physics, Faculty of Physics, Astronomy and Informatics, Nicolaus Copernicus University, Grudziadzka 5, 87-100 Torun, Poland; ^2^Faculty of Biology and Biotechnology, Ruhr-University Bochum, D-44780 Bochum, Germany

## Abstract

We investigate metal-enhanced fluorescence of peridinin-chlorophyll protein coupled to silver nanowires using optical microscopy combined with spectrally and time-resolved fluorescence techniques. In particular we study two different sample geometries: first, in which the light-harvesting complexes are deposited onto silver nanowires, and second, where solution of both nanostructures are mixed prior deposition on a substrate. The results indicate that for the peridinin-chlorophyll complexes placed in the vicinity of the silver nanowires we observe higher intensities of fluorescence emission as compared to the reference sample, where no nanowires are present. Enhancement factors estimated for the sample where the light-harvesting complexes are mixed together with the silver nanowires prior deposition on a substrate are generally larger in comparison to the other geometry of a hybrid nanostructure. While fluorescence spectra are identical both in terms of overall shape and maximum wavelength for peridinin-chlorophyll-protein complexes both isolated and coupled to metallic nanostructures, we conclude that interaction with plasmon excitations in the latter remains neutral to the functionality of the biological system. Fluorescence transients measured for the PCP complexes coupled to the silver nanowires indicate shortening of the fluorescence lifetime pointing towards modifications of radiative rate due to plasmonic interactions. Our results can be applied for developing ways to plasmonically control the light-harvesting capability of photosynthetic complexes.

## 1. Introduction

Metal-enhanced fluorescence (MEF) is a process where emission intensity of a fluorophore is increased via interaction with nearby placed metallic nanoparticles [1–5]. The increase of the emission intensity can be caused either by enhancement of absorption rate or enhancement of the radiative rate of the fluorophore, as well as by a combination of both processes. This effect has been in recent years observed for a variety of systems including organic dyes [6–9], semiconductor nanocrystals [10–14], conjugated polymers [15–17], DNA [18], and naturally evolved biological complexes [19–21]. One of the most important requirements for achieving strong enhancement of the fluorescence intensity concerns is spectral matching between the plasmonic nanoparticles and fluorophores, while at the same time the strength of the interaction depends on the separation between the two components. Detailed work carried out on a single molecule level as well as on ensemble revealed that the fluorescence enhancement is the strongest for the distances in the range of 10–25 nm [22–24]. For thinner spacers nonradiative energy transfer from the fluorophore to the metallic nanoparticle starts to dominate the overall behavior, leading to strong quenching of the fluorescence [25–28]. 

In this work we describe results of optical microscopy and spectroscopy obtained for a light-harvesting complex from algae, peridinin-chlorophyll protein (PCP) coupled to silver nanowires. The PCP complex has been studied in recent years in the context of probing plasmonic interactions in biological systems [29–31]. It is highly applicable to such purposes due to structural simplicity as compared to other photosynthetic complexes, solubility in water, and strong absorption of light in the visible spectral range due to the presence of peridinins [19]. The PCP complex can also be efficiently reconstituted with various chlorophyll mixtures, and such structures have been recently applied for observing influence of plasmonic interactions upon the energy transfer dynamics between two energetically distinguishable chlorophylls embedded in a PCP protein [31]. It has also been shown that for single PCP complexes deposited on a silver island film (SIF) the fluorescence emission can be strongly enhanced but only partially due to an increase of absorption rate. On the other hand, for a structure where the PCP complexes were separated by a 25 nm-thick silica spacer from a SIF layer, the enhancement has been shown to originate almost exclusively from the increase of the absorption rate. Analogous experiments carried out on other photosynthetic complexes coupled to various metallic nanoparticles have confirmed the general idea that it is indeed possible to change the optical properties of functional photosynthetic complexes through coupling with plasmonic excitations in metallic nanoparticles. At the same time, precise understanding of all the factors influencing the strength of the interaction such as geometry of a hybrid nanostructure or application of particular type of metallic nanoparticles has not yet reached the level that would be sufficient for designing devices with tailored properties. 

In this regard, studying the interaction between light-harvesting complexes and metallic nanowires, that can have lengths of tens of micrometers, is particularly interesting. Being long enough for direct imaging using standard microscopy, the nanowires are still plasmonically active as their diameters are often below 200 nm. The plasmon resonance of the silver nanowires appears at around 400 nm with a broad tail towards longer wavelength. Therefore these metallic nanostructures can provide absorption enhancement over broad spectral range that would not be reached with other metallic nanoparticles. Moreover, the ability to image the nanowires using confocal or wide-field microscopy enables direct correlation between the morphology of the sample and the corresponding fluorescence pattern. From the more application-oriented approach, the metallic nanowires are also highly attractive as they can form a conductive network when embedded in polymer films. In this way it is possible to fabricate transparent electrodes for solar cells [32].

The results described in this work focus on understanding mechanisms of metal-enhanced fluorescence in hybrid nanostructures composed of the PCP complexes and silver nanowires. Fluorescence images obtained using wide-field microscopy indicate a strong increase of the emission intensity for the PCP complexes placed in the vicinity of the silver nanowires. The intensity measured at the ends of the nanowires is significantly higher as compared to the PCP complexes placed along the wire. In connection to these results we discuss the influence of sample concentration on the estimated enhancement factors. Combining wide-field microscopy with confocal approach coupled with time-resolved fluorescence measurement yields evidence that increase of the radiative rate contributes substantially towards the total fluorescence enhancement in such a structure.

## 2. Materials and Methods

Peridinin-chlorophyll protein (PCP) is a water-soluble, light-harvesting complex from the dinoflagellate *Amphidinium carterae*. In this work we use the PCP complexes reconstituted with chlorophyll *a*, prepared as described earlier [33]. The concentration of the stock solution was 2 mg/mL. Silver nanowires (AgNWs) were synthesized using polyol process in the presence of ethylene glycol, copper seeds, and poly(vinyl pyrrolidone) polymer. In the reaction ethylene glycol served as reducing reagent and solvent [34]. The morphology of the AgNWs was studied using scanning electron microscopy, while the optical properties of both metallic nanoparticles and light-harvesting complexes in solutions were checked using absorption and fluorescence spectroscopy.

Scanning electron microscopy studies were carried out with a Zeiss AURIGA microscope equipped with in-lens detector. The microscope was operating at 5 kV with a current of 89 pA. A solution of silver nanowires for SEM studies was diluted in pure water and then deposited on GaAs substrates as small droplets. All samples were allowed to dry in ambient conditions. Absorption spectra were recorded on a Varian Cary 50 UV-Vis spectrophotometer. Steady-state fluorescence measurements were performed using a Fluorolog-3 spectrofluorometer (Jobin Yvon) equipped with a double grating monochromator. A Xenon lamp source was used for excitation, and the signal was detected with a thermoelectrically cooled photomultiplier tube with a dark current less than 100 cps.

Hybrid nanostructures composed of silver nanowires and pigment-protein complexes were fabricated on glass coverslips in two different ways using a spin-coating technique. In the first approach we spin-coated 30 *μ*L AgNWs, and then a layer of 20 *μ*L PCP in 2% polyvinyl alcohol (PVA from Sigma-Aldrich) aqueous solution was deposited. Second way of sample preparation was based on spin-coating a layer of 30 *μ*L mixed solution of PCP (15 *μ*L) and AgNWs (15 *μ*L) diluted in 1% aqueous solution of PVA. In order to distinguish both structures we call them layer-by-layer and mixed samples, respectively. In addition, a reference sample was fabricated by spin-coating 30 *μ*L PCP in aqueous PVA solution. During sample preparation particular care was taken to avoid incorporation of any impurities, for all dilutions we used high-purity water (Fluka), and the glass cover slips on which the solutions were spin-coated were cleaned thoroughly using Hellmanex.

Fluorescence intensity maps were measured using a Nikon Eclipse Ti inverted wide-field microscope equipped with an Andor iXon Du-888 EMCCD detector cooled to −75°C for low dark current values. Due to strong plasmon resonance of the AgNWs near 400 nm and the PCP absorption maximum near 480 nm, we used LEDs illuminators with the wavelengths of 405 nm and 480 nm as excitation sources. In order to spectrally narrow the excitation light we used appropriate band-pass filters (FB405-10 and FB480-10). The excitation was then reflected up to the microscope's objective lens (Plan Apo, 100x, oil immersion, Nikon) by a dichroic beam splitter (Semrock FF458-Di02, Chroma 505DCXR). The excitation power after the dichroic mirror was 145 *μ*W and 71 *μ*W for 405 nm and 480 nm excitation wavelengths, respectively. Fluorescence of the PCP complexes was filtered using a narrow-band pass filter (Chroma HQ675-20). Typical parameters used in the experiment were acquisition time of 5 s, electron multiplying gain of 0 and 50 for the higher and lower PCP concentration, respectively, and the size of fluorescence images of approximately 90 × 90 *μ*m.

Spectrally and time-resolved measurements were performed using home-built scanning confocal/wide-field microscope described in detail previously [35]. In the first step, we illuminated the sample surface with a defocused laser beam and collected wide-field fluorescence image. The size of the image was approximately 50 × 50 *μ*m. In this way we were able to localize silver nanowires on the sample surface. In the second step, by switching into the confocal configuration, we positioned a laser spot at a specific location, that is, at the nanowire (either at the end or along the nanowire) or outside the nanowire. In these locations we measured emission spectra. In the third step, we collected fluorescence transients at locations corresponding to these from which the emission spectra were taken.

The excitation laser was focused onto a sample surface using an Olympus long working distance microscope objective LMPlan 50x with the numerical aperture of 0.5. The fluorescence images were collected using an Andor Newton EMCCD DU970N detector equipped with a band-pass filter HQ675-20. The emission spectra were detected using an Amici prism coupled with a CCD detector (Andor iDus DV 420A-BV). Fluorescence transients were measured in a back-scattering geometry using time-correlated single photon counting (TCSPC) as described previously [35]. For the excitation, 30 ps lasers were used with the wavelengths 405 nm and 485 nm, each operating at 50 MHz repetition rate and the excitation power 30 *μ*W and 60 *μ*W for the 405 nm and 485 nm excitation wavelength, respectively. Fluorescence decay curves were detected using a narrow filter D670-10 (Chroma). In order to avoid a fast photo-bleaching process in studied protein-pigment complexes, time-resolved measurements were performed under vacuum conditions with the sample placed in a vacuum chamber. 

## 3. Results and Discussion

**Figure 1:**
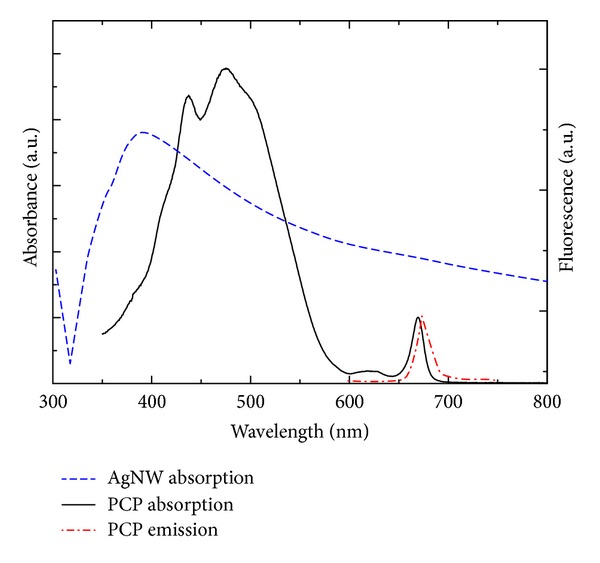
Extinction spectrum of silver nanowires compared with the absorption and emission of the PCP complexes at room temperature. All spectra were measured in solution, and the excitation wavelength for collecting fluorescence was 480 nm.

The PCP is a light-harvesting complex, and its native form contains two chlorophyll *a* (Chl *a*) and eight peridinin (Per) molecules, as determined using X-ray crystallography with 1.3 A resolution [36]. The Chl *a* molecules absorb light in two spectral regions, from 350 to 440 nm and from 600 to 670 nm (as shown in Figure 1). The major light-harvesting pigment in the PCP complex is however Per, which absorbs light from 450 nm to 550 nm. The energy absorbed by Per is then efficiently transferred to the Chls [37]. The fluorescence emission of the PCP complexes comes from weakly coupled Chl molecules and features a narrow band with a maximum at 673 nm (as shown in Figure 1). Silver nanowires feature strong plasmon resonance around 400 nm (shown in Figure 1). The absorption and emission spectra of the PCP overlap with the plasmon resonance of the AgNWs; thus strong interaction between the two nanostructures is expected in a hybrid system. 

**Figure 2:**
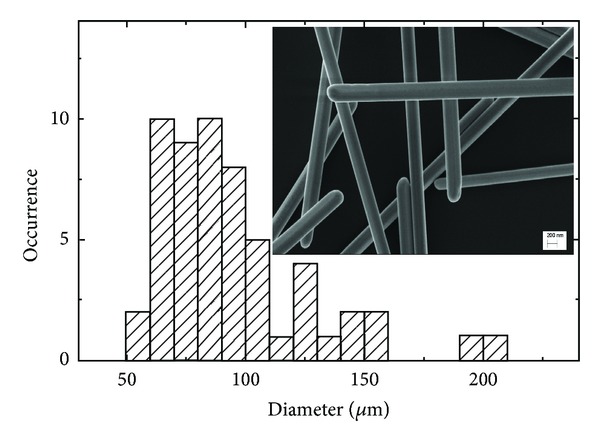
Histogram of the diameters of AgNWs determined from scanning electron microscopy images, example of which is displayed in the inset.

Morphology of the silver nanowires was characterized also using scanning electron microscopy (SEM). A typical SEM image of AgNWs deposited on a substrate is shown in the inset to Figure 2. The nanowires are very long, the diameter is uniform along the length, and we find no tapering or any effect of the kind. Importantly, the nanowires feature sharp ends that can be a source of additional electromagnetic field enhancement due to antenna-like concentration effect. In order to analyze the morphology of the silver nanowires in more detail, we determined diameters of approximately 60 nanowires from the SEM data. The result of the analysis is displayed in Figure 2. The average diameter of the AgNWs used in the experiment is about 95 nm, and the values range from 55 nm to 200 nm. The length of the nanowires varies from 4 *μ*m to 30 *μ*m. 

**Figure 3:**
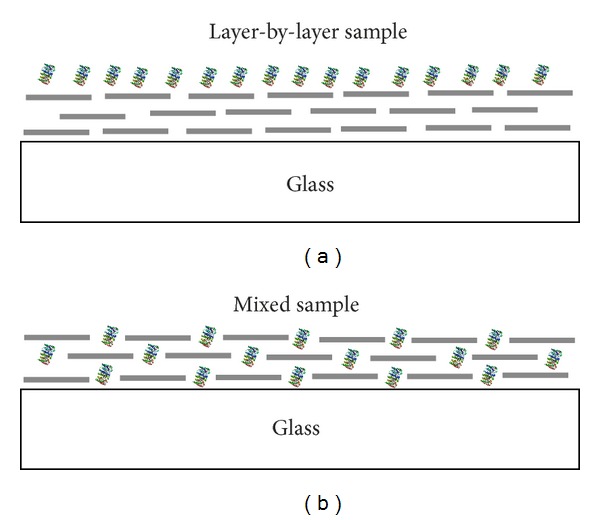
Geometries of hybrid nanostructures comprising the PCP complexes and silver nanowires studied in this work: (a) lbl (layer-by-layer) and (b) mixed structure. Dashed lines:AgNWs; colored objects:PCP complexes. The scale is not conserved.

The effect of metal-enhanced fluorescence in assemblies composed of silver nanowires and the PCP complexes was studied for two sample geometries shown schematically in Figure 3. For the first sample, the layer-by-layer one (Figure 3(a)), which was prepared by spin-coating a layer of PCP complexes on a previously prepared layer of AgNWs, we expect on average longer distance between the PCP complexes and the metallic nanostructures. Also, the absolute number of the PCP complexes interacting with the silver nanowires should be relatively small and restrictred to the nanostructures placed only close to the interface between the two layers. On the other hand, for the mixed sample (Figure 3(b)), fabricated by mixing solutions of PCP complexes and silver NWs and spin-coating the mixture on a glass coverslip, we expect that larger fraction of the PCP complexes should be located close enough to the nanowires to facilitate strong plasmonic interactions between the two components. The two architectures were chosen as they are most commonly suggested as possible ways to improve efficiency of plasmonically enhanced solar cells [32]. 

**Figure 4:**
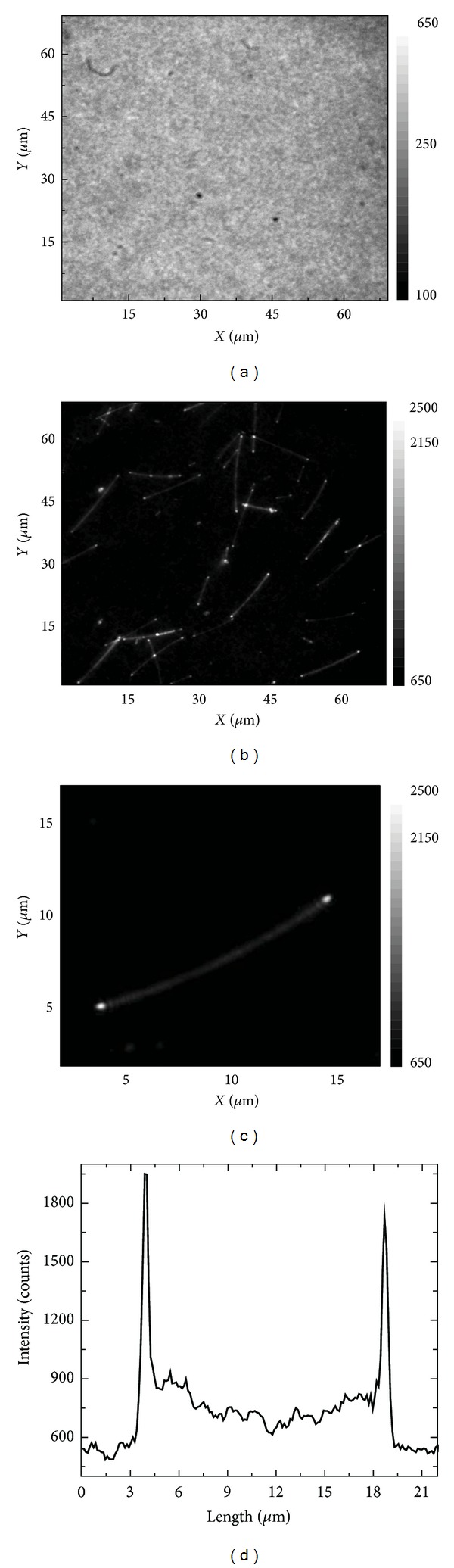
Fluorescence intensity maps obtained using wide-field microscopy for the PCP complexes excited with 480 nm light. (a) Reference sample with PCP complexes only, (b) PCP complexes mixed with silver nanowires, (c) zoom into a single, well-separated AgNW, and (d) emission intensity distribution along a single AgNW.

In Figure 4 we summarize major findings of the fluorescence imaging using wide-field microscopy for the mixed structure. A large-area fluorescence image obtained under 480 nm excitation is shown in Figure 4(b). In contrast to corresponding fluorescence image collected for the reference sample, where only PCP complexes were present (Figure 4(a)) and which features highly uniform intensity pattern, the image obtained for the sample containing AgNWs is highly inhomogeneous. These inhomogeneities have very well-defined pattern. Namely, we observe larger number of randomly distributed elongated areas of increased fluorescence intensity. Comparison of the fluorescence image with a transmission image (not shown) measured for the same area of the sample strongly suggests that these elongated shapes seen in the fluorescence image correspond to the positions, where also silver nanowires are present. Therefore, we conclude that the enhanced emission originates from the PCP complexes placed in the vicinity of the silver nanowires. The presence of enhanced emission of the PCP complexes along the silver nanowires indicates that there is significant number of the PCP complexes placed close enough to the nanowires to have their absorption/emission affected. At the same time, however, this result suggests that the PCP complexes are far enough to diminish the negative influence of the nonradiative energy transfer to the silver nanowires that would result in efficient quenching of the fluorescence emission. It is important to note also that there are many PCP complexes not coupled to the AgNWs; therefore, the intensity measured away from the nanowires is finite. In addition, due to approximately 1 *μ*m thick polymer layer containing both the PCP complexes and the AgNWs, there are also PCP complexes isolated from the nanowires in a vertical direction. This fact is important for estimating the actual enhancement factor from the wide-field fluorescence microscopy. A fluorescence image obtained for a single, well-isolated AgNW is shown in Figure 4(c) in order to demonstrate that the intensity of the fluorescence emission is significantly higher at the ends of the nanowire as compared to the locations along the nanowire. A typical intensity profile measured for such a nanowire is displayed in Figure 4(d), where we plot the dependence of the PCP emission intensity on the position along the silver nanowire. For this particular nanowire we observe sharp and spatially narrow peaks at the ends of the nanowire with the emission intensities approximately three times higher than the intensity measured for the PCP complexes off the nanowire and along the nanowire. Over 90% of isolated nanowires feature such a behavior. In the case of nanowires that touch each other the images are more complex with unspecific hot spots featuring extremely large values of the fluorescence intensity being observed. The appearance of much higher fluorescence intensity at the ends of the nanowires seems to be a generic effect, independent of the excitation wavelength. Final qualitative observation related to the results presented in Figure 4 concerns apparent existence of an oscillatory pattern (Figure 4(c)) of the fluorescence intensity from the ends of the nanowire towards the middle of it. The explanation of this effect is still unclear, and more detailed analysis corroborated with theoretical modelling is required. Nevertheless, such an oscillatory pattern could point towards unique plasmonic properties of the silver nanowires.

In order to analyze the influence of plasmon excitations in silver nanowires on the optical properties of the PCP complexes we determined fluorescence intensity for three classes of the PCP complexes: those located at the ends of nanowires, those placed along the nanowires, and those that were off the nanowires. The analysis was carried out for series of images similar to the one shown in Figure 4, and for that purpose we chose only single and well-separated NWs. In this way we ignore situations when the nanowires are crossed or connected with each other, where artefacts associated with even stronger fluorescence signals were observed, presumably due to formation of hot spots. The procedure was different for estimating the fluorescence intensity for the PCP complexes located along the NWs and for those at the ends of the NWs. In the first case we took the value of the emission intensity calculated as an average fluorescence intensity obtained from all pixels along a line that followed the individual shape of each AgNW. The reference intensity, used for calculating the fluorescence enhancement factor for the particular AgNW, was obtained by placing the identical line in the vicinity of that of the NW, but where no enhanced emission of the PCP complexes was observed. In this way the analysis is less sensitive to possible inhomogeneities of the emission intensity along the nanowire, as well as to slight differences of the illumination intensity in the wide-field fluorescence imaging experiment. For that part of the analysis the pronounced bright spots at the ends of the nanowires were omitted. In these cases, the estimation of the enhancement factor was based on taking the emission intensity value corresponding to the brightest spot detected in this region and comparing it to the average intensity of the PCP emission aside from any given nanowire. Identical approach was applied for the results obtained for the two types of hybrid nanostructure studied and for both excitation wavelengths. 

**Figure 5:**
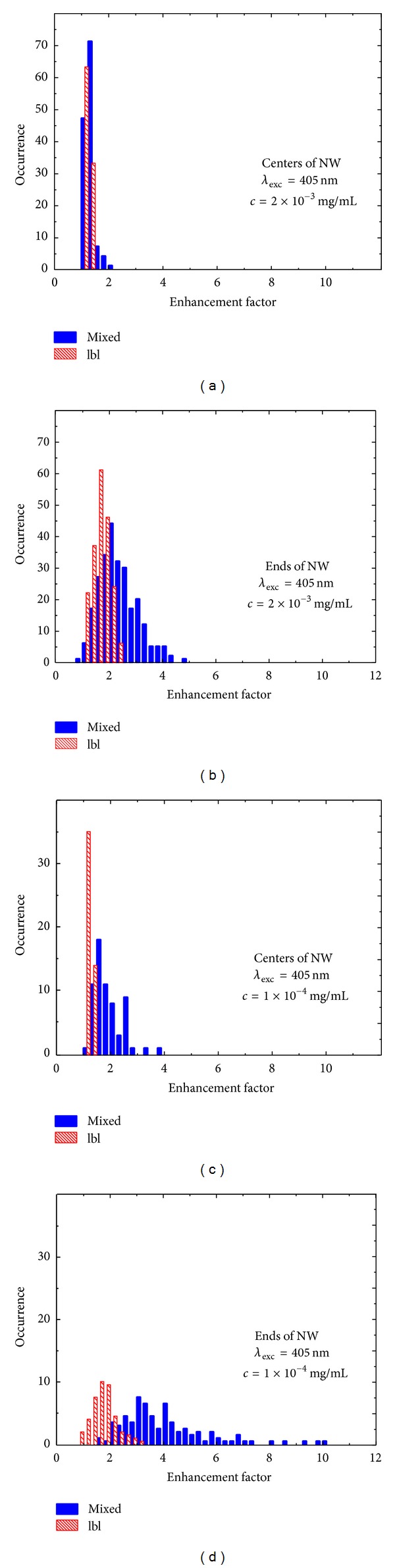
Histograms of the enhancement factors determined for the mixed (solid bars) and layer-by-layer (dashed bars) samples with the PCP complexes excited at 405 nm. Data shown in (a) and (b) correspond to the PCP concentration of 2 × 10^−3^ mg/mL and for the central parts of NW and for the ends of NW, respectively. Data shown in (c) and (d) correspond to the PCP concentration of 1 × 10^−4^ mg/mL and for the central parts of NW and for the ends of NW, respectively.

The calculated emission enhancement factors obtained using wide-field fluorescence microscopy of the PCP complexes coupled to AgNWs are displayed in Figure 5 for the excitation wavelength of 405 nm for both the mixed (solid bars) and the layer-by-layer (dashed bars) samples. In addition, we include the enhancement factors determined for two different concentrations of the PCP complexes. The excitation wavelength of 405 nm is essentially resonant with the plasmon resonance of the nanowires. Right panels show enhancement factors obtained for the PCP located along the NWs: (a) for 2 × 10^−3^ mg/mL PCP concentration and (c) for 1 × 10^−4^ mg/mL PCP concentration. Left panels show distributions of calculated enhancement factors obtained for the ends of NWs: (b) for the 2 × 10^−3^ mg/mL concentration of PCP complexes and (d) for the 1 × 10^−4^ mg/mL concentration of the PCP complexes. For the PCP concentration of 2 × 10^−3^ mg/mL in the mixed sample, the values of enhancement factor for the PCP complexes located along the silver nanowires vary from 1.05 to 2.02, while for the layer-by-layer sample those values vary from 1.03 to 1.42. The enhancement in this case is relatively low. In contrast, enhancements calculated for the same PCP concentration but at the ends of NWs vary from 0.95 to 6.63 for the mixed sample and from 1.01 to 2.42 for the layer-by-layer sample. The average enhancement factors resulting from the coupling between PCP complexes and plasmonic excitations in the AgNWs for the mixed (layer-by-layer) sample geometry are found to be 1.32 (1.22) and 2.40 (1.67) along the nanowires and at the ends of the nanowires, respectively. The differences between enhancement factors determined for both sample geometries are better visible in the case of the structure with lower PCP concentration (lower row in Figure 5). In this case the values of enhancement factor for the mixed (layer-by-layer) sample vary from 1.12 (1.08) to 3.86 (1.36) and from 1.57 (0.94) to 13.36 (3.17) as calculated along the silver nanowires and at ends of the nanowires, respectively. Thereupon the average enhancement factors for the mixed (layer-by-layer) sample are 1.93 (1.22) and 4.11 (1.73) for the PCP complexes placed along the NWs and at the ends of NWs, respectively.

**Figure 6:**
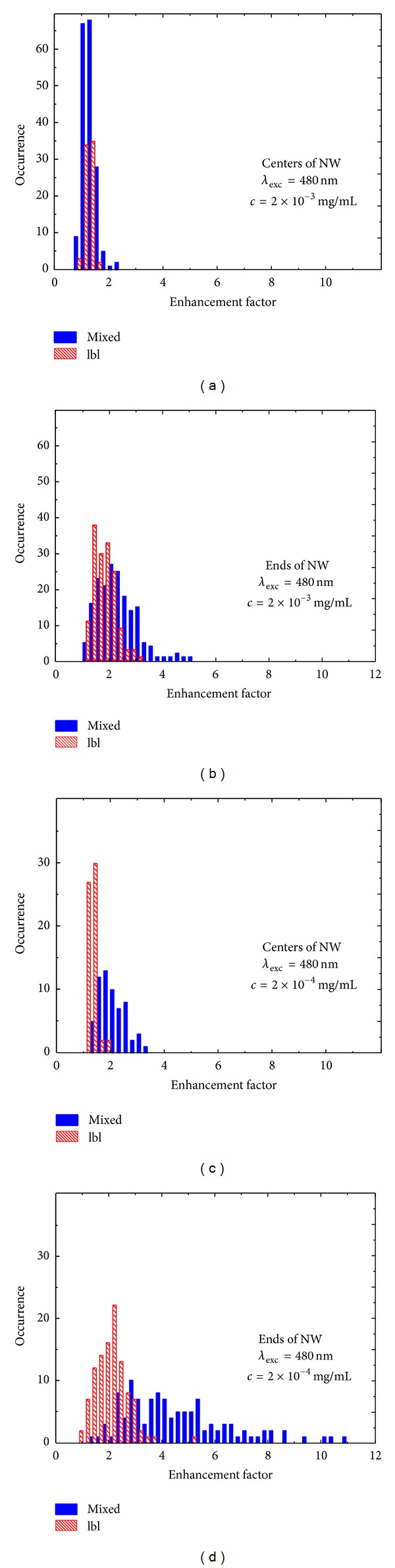
Histograms of the enhancement factors determined for the mixed (solid bars) and layer-by-layer (dashed bars) samples with the PCP complexes excited at 480 nm. Data shown in (a) and (b) correspond to the PCP concentration of 2 × 10^−3^ mg/mL and for the central parts of NW and for the ends of NW, respectively. Data shown in (c) and (d) correspond to the PCP concentration of 1 × 10^−4^ mg/mL and for the central parts of NW and for the ends of NW, respectively.

Similar analysis as described above was done for the intensity maps of the PCP fluorescence collected with the excitation at 480 nm, where the PCP complex features the strongest absorption. The results are displayed in a similar manner as for the 405 nm excitation, in Figure 6 with the same legend and color coding applied. Right panels show enhancement factors obtained for the PCP located along the NWs: (a) for 2 × 10^−3^ mg/mL PCP concentration and (c) for 1 × 10^−4^ mg/mL PCP concentration. Left panels show distributions of calculated enhancement factors obtained at the ends of the NWs: (b) for the 2 × 10^−3^ mg/mL concentration of PCP complexes and (d) for the 1 × 10^−4^ mg/mL concentration of the PCP complexes. For the higher concentration of the PCP complexes and for the mixed sample, the values of enhancement factors for the PCP complexes placed along the nanowires vary from 0.83 to 2.44, while for the layer-by-layer sample those values vary from 0.89 to 1.75. In the case of enhancement factor obtained at the ends of NWs the values vary from 1.10 to 5.02 and from 1.03 to 3.04 for the mixed and the layer-by-layer sample, respectively. The average enhancement factors for the mixed (layer-by-layer) sample are 1.35 (1.25) and 2.32 (1.76) along the nanowires and at the ends of the nanowires, respectively. Similarly as in the case of 405 nm excitation, the obtained values of the enhancement factor depend upon the concentration of the PCP complexes used for sample preparation. Namely, for the PCP concentration of 1 × 10^−4^ mg/mL, the values of enhancement factors for the mixed (layer-by-layer) sample vary from 1.44 (1.12) to 3.39 (1.77) and from 1.43 (0.90) to 19.13 (5.11) as calculated for the PCP complexes placed along the NWs and at the ends of the NWs, respectively. Consequently, the average enhancement factors for the mixed (layer-by-layer) samples are found to be 2.10 (1.27) and 4.83 (2.04) for the PCP located along the NWs and at the ends of the NWs, respectively. 

We derive several important conclusions from the analysis of the wide-field fluorescence microscopy images measured for the PCP complexes coupled to the AgNWs. First, we observe fluorescence enhancement for the PCP complexes located in the vicinity of the silver nanowires and, furthermore, the emission enhancement is significantly stronger when the PCP complexes are placed at the ends of the silver nanowires in comparison to the PCP complexes located along the nanowires. Moreover, the average values of the enhancement factor obtained for the hybrid nanostructures excited at 405 nm, which is resonant with the plasmon resonance maximum of the nanowires, are similar to the enhancement factors obtained for 480 nm excitation, which is the maximum of the PCP absorption band. On the other hand, we observe strong dependence of the measured enhancement factors on the geometry of the structure and the concentration of the PCP complexes, in particular in the case of the mixed samples. The fluorescence enhancement for samples with lower concentration of the PCP complexes (1 × 10^−4^ mg/mL) is stronger than in the case of the samples with higher concentration of the PCP complexes (2 × 10^−3^ mg/mL). Last but not least, much broader distribution of the enhancement factors is extracted in the case of PCP complexes placed at the ends of the AgNWs than for the PCP localized along the NWs, in particular for the lower PCP concentration. 

The method used for analyzing the wide-field microscopy fluorescence images of the PCP complexes coupled to the silver nanowires has only qualitative and approximate character. It is due to the sample preparation method and the ratio between the number of the PCP complexes probed in the experiment and the number of the PCP complexes coupled to the AgNWs. The sample preparation through spin-coating technique results in films with a thickness of several hundred nanometers. On the other hand, it is well known that plasmon-induced fluorescence enhancement requires that the fluorophore is placed at distances between 10 nm and 30 nm from the metallic nanoparticles. Consequently, in the case of the wide-field fluorescence imaging experiment, where the focal spot of the excitation beam has a length of about 600 nm, the contribution of the PCP complexes that are strongly coupled to the AgNWs is overshadowed by the fluorescence measured for the complexes isolated from the AgNWs. The actual ratio between the two subsets of the PCP complexes is a function of the polymer layer (the thinner it is, the stronger the contribution of the coupled PCP complexes to the total emission intensity measured in the experiment is). Thereby, in the wide-field microscopy experiment, the enhancement factors are always underestimated. 

Consideration of relative contributions of the PCP complexes coupled and uncoupled to silver nanowires allows for interpreting distributions of the enhancement factor displayed in Figures 5 and 6. In the case of the layer-by-layer sample the enhancement factors show no dependence upon the concentration of the PCP complexes. Such a behavior is expected as the ratio between the PCP complexes coupled and isolated from the silver nanowires should not change with changed concentration of the PCP complexes in a layer deposited onto a layer of silver nanowires. The distributions measured for the layer-by layer sample are also quite homogeneous, presumably since the variation in the coupling strength takes place essentially in one dimension in the direction perpendicular to the structure. In contrast, in the case of the mixed sample, the distributions of the enhancement factors are significantly broader, probably due to higher number of the degrees of freedom for determining distances and orientations between the PCP complexes and the silver nanowires. In addition, substantial increase of both the average and maximum enhancement factors for the mixed sample prepared with lower concentration of the PCP complexes suggests that during mixing there should be some sort of unspecific interaction between the two types of the nanostructures, as apprently the number of the PCP complexes interacting with the silver nanowires is increased. While the results of wide-field fluorescence imaging provide an excellent way to demonstrate metal-enhanced fluorescence in plasmonic hybrid nanostructures, more quantitative approaches are required in order to understand these effects in greater detail. 

**Figure 7:**
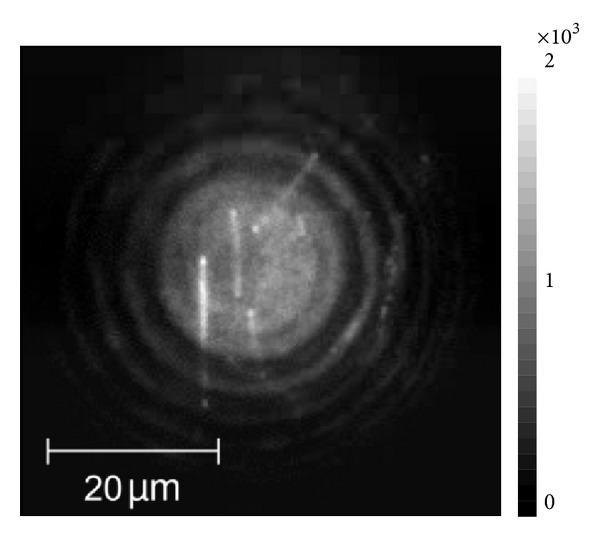
Wide-field fluorescence image of the PCP complexes mixed with AgNWs. The excitation wavelength and PCP concentration were *λ*_exc_ = 405 nm and 2 × 10^−3^ mg/mL, respectively.

In Figure 7 we show a wide-field fluorescence image of the hybrid nanostructure containing the PCP complexes and silver nanowires. Qualitatively the image is very similar to the data described previously; we can determine the positions of the nanowires and observe strong increase of the fluorescence emission of the PCP complexes placed in the vicinity of the nanowires. Also, the fluorescence intensity measured at the ends of the nanowires is stronger as compared to areas along the nanowires, in full agreement with previous results. In the microscope configuration used to measure this image we are able to switch to a confocal configuration and collect fluorescence spectra and decays for any given location across the sample surface. 

**Figure 8:**
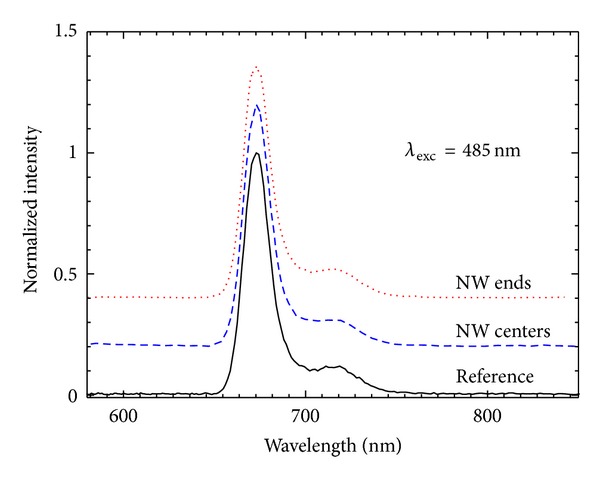
Fluorescence spectra of PCP complexes deposited on Ag nanowires measured for the mixed sample under excitation wavelength of *λ*_exc_ = 485 nm. Data obtained for the laser spot placed at the end of the nanowire (dotted line), along the nanowire (dashed line), and off the nanowire (solid line).

Examples of fluorescence spectra collected for a laser beam placed along the nanowire and at the end of it are displayed in Figure 8, where for comparison we also included a spectrum measured off the nanowires. The spectra were shifted vertically for clarity. Importantly, in all cases the shape and maximum emission wavelength of the fluorescence emission are identical. This result indicates that PCP complexes remain intact upon coupling with the silver nanowires, and that the functionality of the photosynthetic complexes in terms of energy transfer is preserved in the hybrid nanostructure. Qualitatively similar result was obtained for the layer-by-layer sample. Demonstrating little or no effect of the proximity of metallic nanoparticles upon the function of the pigment-protein complex is vital, as sometimes it has been shown that coupling between metallic layers and photosynthetic complexes results in modifications of the emission spectra, thus the protein function [20]. We note that for our samples less than 1% of all measured fluorescence spectra had different shapes than the ones displayed in Figure 8.

**Figure 9:**
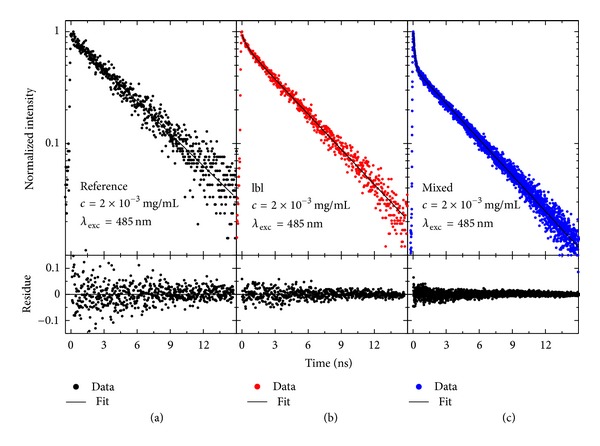
Typical fluorescence decay curves of (a) the reference and for hybrid nanostructures that contain the PCP complexes deposited on Ag nanowires prepared using (b) layer-by-layer and (c) mixed approaches. The samples were excited at *λ*_exc_ = 485 nm.

Fluorescence transients were collected subsequently to measuring the emission spectra only for these locations, where the spectra were similar to the ones shown in Figure 8. Examples of decay curves measured for the layer-by-layer and mixed samples are shown in Figure 9. The transients obtained for the hybrid nanostructures are compared with the one collected for the reference—PCP-only—sample. The points represent the data while the lines are fits, either single exponential for the reference or biexponential for the hybrid nanostructures. Residue graphs attached below each decay indicate very good quality of the fitting procedure. In this way we obtained decay constants and amplitudes for approximately twenty nanowires for each sample. In the case of the reference sample, the PCP complexes feature monoexponentially with a typical decay constant of about 4 ns, in agreement with previously published results [30]. In contrast, for the PCP complexes located along the silver nanowires in the mixed sample we obtained two decay times, the longer one comparable to the reference sample and the shorter one equal to approximately 0.3 ns for both excitation wavelengths of 405 nm and 480 nm. On the other hand, in the case of the layer-by-layer sample the longer time was again comparable to the reference, while the fast decay can be approximated with constants of about 0.2 ns and 0.5 ns for 405 nm and 485 nm excitations, respectively. Interestingly, in the case of the mixed sample the amplitude associated with the short decay constant is about twice as much as in the case of the layer-by-layer sample. This suggests that the number of the PCP complexes interacting with the silver nanowires is larger in the former. 

**Figure 10:**
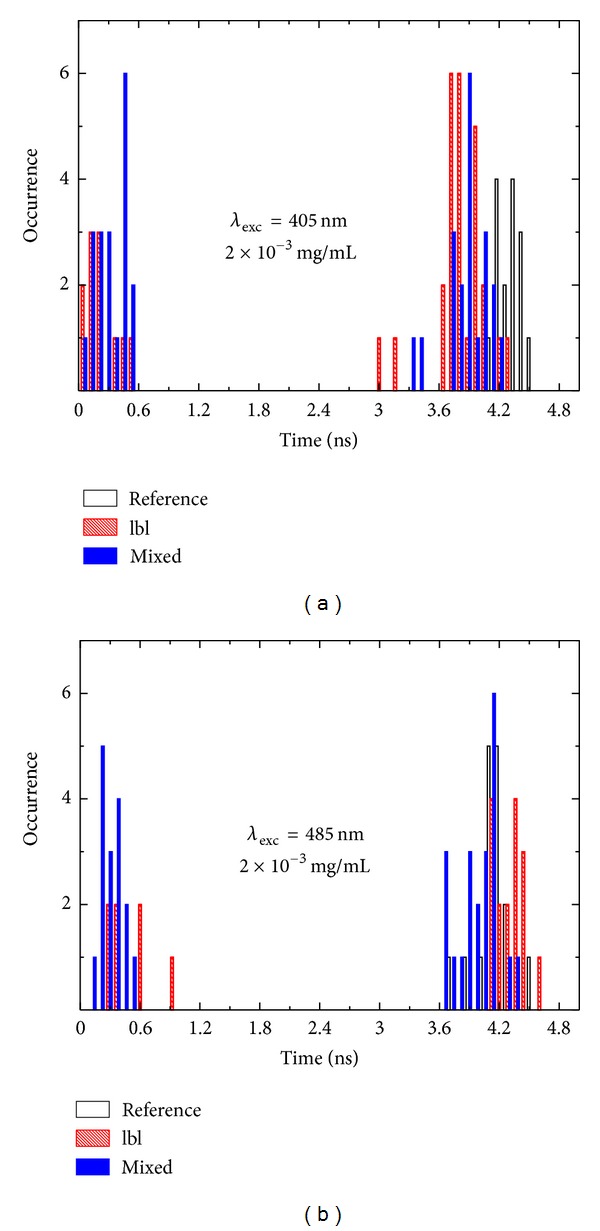
Histograms of fluorescence lifetimes of PCP complexes deposited on Ag nanowires for the layer-by-layer (red bars) and mixed (blue bars) samples. The results obtained for hybrid nanostructures are compared with the PCP-only reference (black bars). Decay times extracted for the excitation wavelength of (a) *λ*_exc_ = 405 nm and (b) *λ*_exc_ = 485 nm are shown.

The decay constants extracted from fluorescence transients measured for approximately 20 nanowires in the mixed and layer-by-layer samples are displayed in Figure 10 for the excitation wavelengths of (a) 405 nm and (b) 485 nm. The appearance of the decay component significantly shorter than that measured for the reference sample is a result of strong plasmon-induced modification of the radiative properties of the chlorophyll molecules embedded in the PCP protein. The long component measured for the laser beam placed onto a nanowire originates from the PCP complexes that are isolated from the nanowire. In fact, since the diameter of the laser spot is about 1 *μ*m as compared to diameters of the AgNWs below 200 nm, it is expected to detect signal associated with the PCP complexes that are far away from the nanowires; thus their radiative properties remain unaffected. On the other hand, the strong reduction of the fluorescence decay time for the PCP complexes coupled to the silver nanowires indicates that plasmon excitations in metallic nanoparticles influence radiative properties of the chlorophylls in the PCP complexes. The reduction is approximately 10-fold, which should result in similar increase of the fluorescence intensity, should no additional plasmon-induced enhancement of the absorption has taken place. Such strong enhancements we observe only in a few cases using wide-field microscopy imaging of the fluorescence of the hybrid nanostructures. This discrepancy is the result of underestimation of the enhancement factors when using only wide-field microscopy imaging of layered samples. This is exactly the reason why it is necessary to include time-resolved spectroscopy for more quantitative analysis of the metal-enhanced fluorescence in plasmonic hybrid nanostructures. On the other hand, single molecule experiments, where only an individual PCP complex can be probed at a time, should provide even a better and more precise way for determining interactions between plasmonically active metallic nanostructures and photosynthetic complexes.

## 4. Conclusions

We observe and analyze metal-enhanced fluorescence of the peridinin-chlorophyll complexes coupled to silver nanowires. Regardless of the sample preparation method we find an increase of the fluorescence intensity for the PCP complexes placed in the vicinity of the silver nanowires with particularly strong effect measured when the PCP complexes are at the ends of the nanowires. Spectrally resolved and time-resolved fluorescence measurements indicate that the emission of the PCP complexes coupled to silver nanowires is not affected by the interaction with plasmon excitations, that points towards preserved functionality of the pigment-protein complex in hybrid nanostructures. The fluorescence decays reveal two decay component, one (~4 ns) which we attribute to uncoupled PCP complexes and another (ranging from 0.2 nm to 0.5 nm), which originates from the PCP complexes strongly interacting with the plasmonic excitations in silver nanowires. Such substantial shortening of the fluorescence decay indicates that increase of the radiative rate plays an important role in our hybrid nanostructures.
